# An unusual case of IgG4-related disease presenting as lower limb fasciitis

**DOI:** 10.1016/j.radcr.2025.12.040

**Published:** 2026-02-20

**Authors:** Jason Kei Chak Mak, Louisa Catherine Firmin, James Roberts, Alexandra Dudek

**Affiliations:** a Department of Radiology, University College London Hospital, London, UK; b Institute of Nuclear Medicine, University College London Hospital, London, UK

**Keywords:** IgG4-related disease, Soft tissue, Fasciitis, Magnetic resonance imaging, ^18^F-FDG-PET/CT

## Abstract

Immunoglobulin G4-related disease (IgG4-RD) is an immune-mediated fibroinflammatory condition that can affect nearly any organ. We report an unusual case of IgG4-RD presenting as gradually progressing bilateral lower limb fasciitis in a 29-year-old male patient. He was initially diagnosed with lower limb cellulitis; however, the symptoms did not improve with long courses of intravenous antibiotics. Autoimmune screen was negative and there were no features to suggest an underlying atypical infection. Subsequent cross-sectional imaging demonstrated deep fasciitis in both lower legs and a deep incision biopsy showed characteristic storiform IgG4 plasma cell tissue infiltration. The patient had an excellent clinical response to steroid and immunosuppressant therapy. IgG4-RD should be considered as a differential diagnosis in a patient presenting with prolonged, unexplained soft tissue swelling, even if there are no other sites of involvement.

## Introduction

Immunoglobulin G4-related disease (IgG4-RD) was first recognised as a distinct systemic condition in 2003 [1] and represents an immune-mediated fibroinflammatory process that can affect virtually any combination of organs, with the pancreas, kidneys, periorbital tissues, salivary/lacrimal glands, and retroperitoneum being classical sites of involvement. Musculoskeletal presentations of IgG4-RD are very rare; there have been several documented reports of soft tissue masses [2,3] but isolated presentation as fasciitis is extremely uncommon. To our knowledge, this patient represents only the second such case in the literature [4].

## Case report

A 29-year-old male patient of South Asian origin presented four times to our hospital over a 2-month period with recurrent lower limb swelling. The patient also had longstanding (≥5 years) iron deficiency anaemia of unclear aetiology. He had never travelled outside of the UK. There was no history of trauma, Raynaud’s syndrome, substance abuse, immunosuppression or malnutrition.

On initial admission, the swelling was confined to the right lower leg, which gradually progressed to involve the entirety of both lower legs from just above the knee to the distal foot (with sparing of the toes) by the third admission. The skin was hyperpigmented. No palpable soft tissue mass, abscess or features of necrotising fasciitis were demonstrated. Muscle power was normal.

Inflammatory marker levels were persistently elevated throughout his admissions, with CRP and ESR measuring up to 159 mg/L and 95 mm/hr respectively. Markedly raised D-dimer levels (up to 3420 µg/I FEU) were also noted, but computed tomography and ultrasonography excluded any arterial or venous thrombosis. Autoimmune screen (including scleroderma, vasculitis, and coeliac disease) was negative, and there was no evidence of any recent atypical or viral infection (including HIV and CMV). Creatine kinase level was normal and echocardiography was negative for cardiac failure or infective endocarditis.

The patient had severe iron deficiency anaemia (Hb down to 38 g/L) that did not fully normalise despite multiple transfusions. B12 and folate levels were normal. There was no evidence of haemolysis or haemoglobinopathy, and endoscopy/colonoscopy was negative for a gastrointestinal bleeding source. Bone marrow biopsy excluded an underlying haematological malignancy but showed increased red cell turnover, likely reactive.

**Fig. 1:**
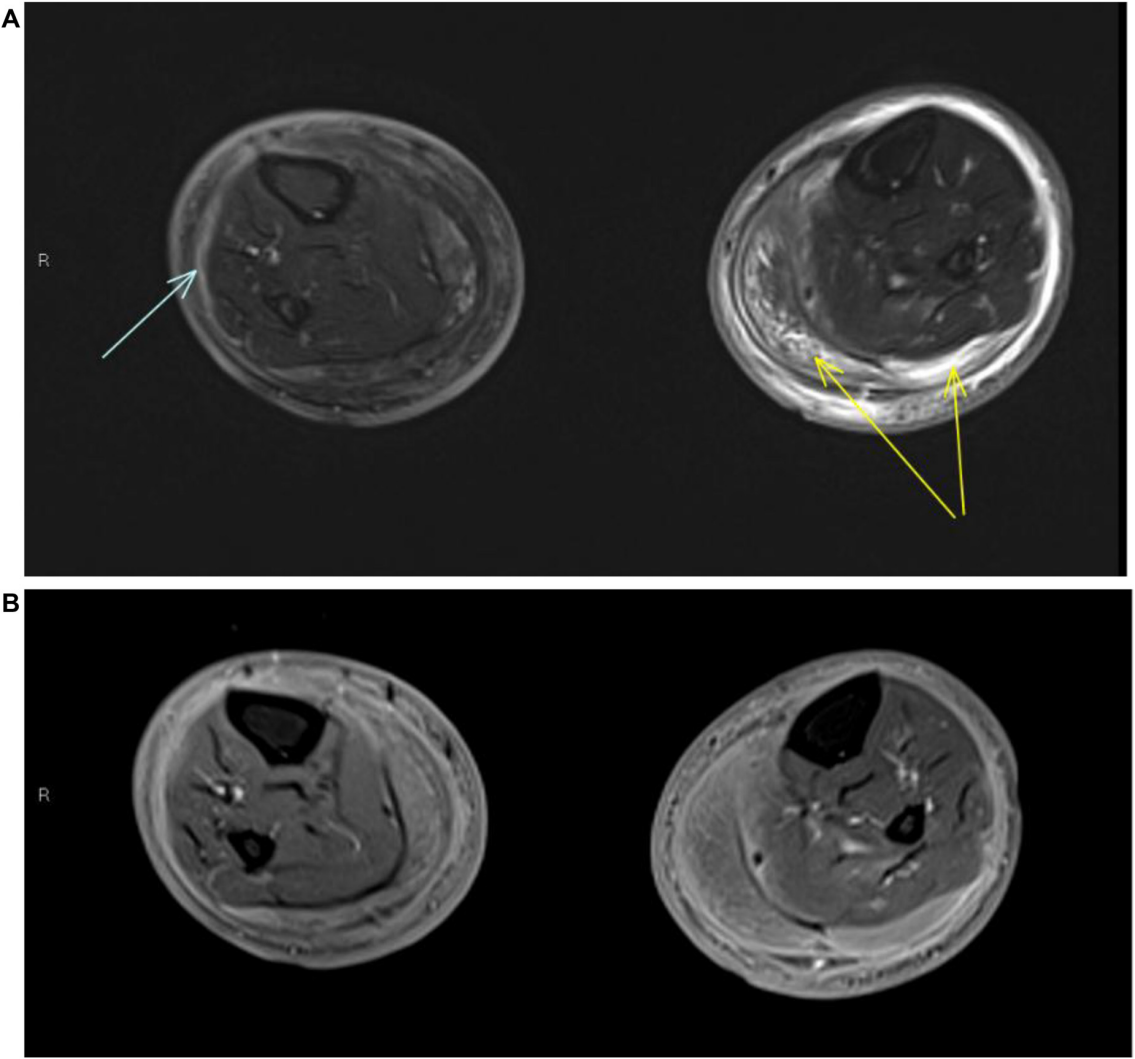
MRI imaging of a 29-year-old male patient with IgG4-related lower limb fasciitis. Axial STIR MRI **(A)** and postcontrast **(B)** images demonstrate circumferential thickening of the deep fascia of both lower legs, in keeping with recurrent fasciitis. Superficial circumferential oedema is seen on the right side while a deeper circumferential layer of subcutaneous oedema is seen on the left. Mild oedema deep to the deep fascia on the right side is suggestive of more acute inflammation (blue arrow). The muscles are largely spared, with localised myositis seen in the left medial and lateral gastrocnemius (yellow arrows). (The upper legs were not imaged due to patient intolerance.) MRI, magnetic resonance imaging; STIR, Short Tau Inversion Recovery; IgG4, immunoglobulin G4.

**Fig. 2:**
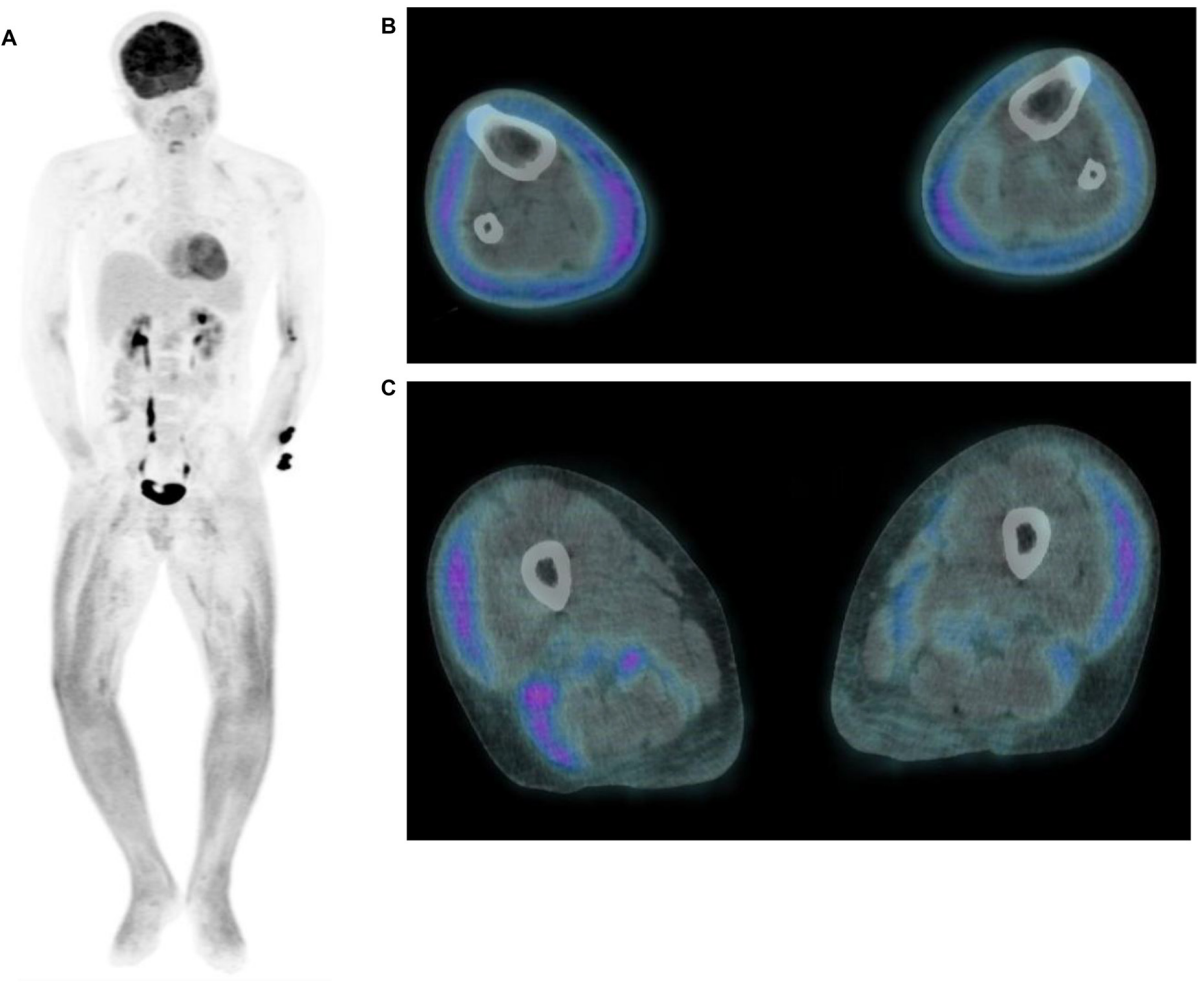
^18^F-FDG-PET/CT imaging of a 29-year-old male patient with IgG4-related lower limb fasciitis. Maximum intensity projection images of the ^18^F-FDG-PET/CT demonstrate diffuse tracer uptake in both lower limbs **(A)**. No classical IgG4 disease sites or any other FDG-avid disease are demonstrated. Fused axial slices of the ^18^F-FDG-PET/CT demonstrate increased avidity at the fascial planes within the anterior and posterior compartments of the lower legs **(B)**; these changes also extend to involve the upper legs **(C)**. ^18^F-FDG-PET/CT, ^18^F-fluorodeoxyglucose-positron emission tomography/computed tomography; IgG4, immunoglobulin G4.

Initially, the leg swelling was felt most likely to be due to cellulitis. However, CRP levels remained elevated (>100 mg/L) despite long courses of intravenous antibiotics and the frequent readmissions prompted further cross-sectional imaging. Magnetic resonance imaging (MRI) demonstrated deep fasciitis in both lower legs [Fig. 1A&B] with corresponding diffuse avidity on ^18^F-fluorodeoxyglucose (^18^F-FDG)-positron emission tomography/computed tomography (PET/CT) [Fig. 2A-C]. No further nonphysiological tracer accumulation was demonstrated, in particular no classical IgG4-RD sites.

Given the diagnostic uncertainty, the patient subsequently underwent a deep incision biopsy of his right anterolateral lower leg, which revealed richly vascular, sheet-like fibroinflammatory proliferation with a storiform pattern that is highly characteristic of IgG4-RD [5]. Immunostaining demonstrated markedly increased IgG4 plasma cell infiltration, with more than 20 IgG4 plasma cells per high powered field and an IgG4/IgG plasma cell ratio exceeding 40%, reinforcing the diagnosis.

Correlation was made with serum IgG4 concentration, which was found to be mildly raised at 1.34 g/L (normal ≤1.30 g/L). Unfortunately, the sample was obtained 6 days after steroid treatment had been initiated, which might have influenced the reading.

The patient responded well to a tapering course of prednisolone followed by mycophenolate mofetil, with resolution of both the leg changes and anaemia (latest Hb 140 g/L). Serum levels of inflammatory markers and IgG4 (1.04 g/L) also normalised after treatment.

## Discussion

The pathophysiology of IgG4-RD remains incompletely understood and its presentation can be highly variable and overlap with other systemic fibroinflammatory conditions; the disease is therefore likely underdiagnosed [6]. In the present case, due to the absence of involvement of classical sites, IgG4-RD was not suspected until the patient’s final admission, leading to a 2-month delay in treatment from initial presentation.

On initial clinical examination, only the superficial subcutaneous layer was thought to be involved, thus prompting the consideration of cellulitis or inflammatory dermatitis. However, cross-sectional imaging showed that the soft tissue changes extended to involve the deep compartment of both lower legs, with MRI and ^18^F-FDG-PET/CT demonstrating features in keeping with a diffuse acute-on-chronic deep fasciitis with relatively mild adjacent myositis.

The patient was apyrexial during all his admissions, and there were no further features to suggest an atypical mycobacterial, fungal or viral infection. There was no convincing autoantibody positivity to suggest an underlying connective tissue disease. Although it can sometimes be difficult to distinguish between fibroinflammatory changes and malignant soft tissue tumours with a fibrous component (e.g. myxofibrosarcoma) on imaging alone, MRI did not demonstrate an infiltrating or focal soft tissue mass.

A particularly important differential diagnosis to exclude in this case is eosinophilic fasciitis, which can present very similarly to IgG4-RD and typically also responds rapidly to steroids. However, serum eosinophil levels were normal and there was no histological evidence of an eosinophilic infiltrate.

Ultimately, despite the initially ambiguous clinical picture, the diagnosis of IgG4-RD was confidently established by the histopathological analysis and immunostaining profile of the deep incision biopsy sample, which demonstrated the characteristic storiform pattern of fibrosis coupled with a predominance of IgG4 subclass plasma cells. Given that the imaging features of IgG4-RD can be very nonspecific, histopathological confirmation of the diagnosis is critical.

The patient’s anaemia never responded adequately to red blood cell transfusions and only normalised after steroid treatment, suggesting that it was secondary to chronic inflammation. Anaemia has been reported in patients with IgG4-RD [3], but in this case there were no features of autoimmune haemolytic anaemia, which is the subtype more typically associated with IgG4-RD [7].

A limitation of our case is that the serum IgG4 concentration on initial presentation was unknown. At the time of measurement, the serum IgG4 level was only mildly elevated and did not meet the diagnostic cut-off value of 1.35 g/L proposed by the Japanese 2020 revised comprehensive diagnostic criteria [8]. However, it must be emphasised that serum IgG4 concentration is not universally considered to be a reliable diagnostic marker of IgG4-RD [9] and would not have altered the final diagnosis established by imaging and histopathological analysis in this case.

In summary, we present a very atypical case of IgG-4-RD presenting as isolated lower leg fasciitis. IgG4-RD should be considered as a differential diagnosis when there is prolonged, unexplained subcutaneous tissue swelling. Whole body imaging (to assess for any involvement of classical sites), in addition to dedicated biochemical and histopathological workup, is required to differentiate IgG4-RD from its many mimickers.

## Patient consent

Written informed consent has been given by the patient for this case and the associated images to be published.
